# Supplementary material for: Out-of-hospital cardiac arrest and ambient air pollution: A dose-effect relationship and an association with OHCA incidence
Source: PLoS One. 2021 Aug 25;16(8):e0256526. doi: 10.1371/journal.pone.0256526 (PMC8386838; doi:10.1371/journal.pone.0256526)
Supplement: S1 Table — (DOCX) [file pone.0256526.s001.docx]

**S1 Table. Summary characteristics for study population with out-of-hospital cardiac arrest.**

| **Variable** | **n= 1582** |
| --- | --- |
| **Male gender (%)** | 906 (57) |
| **Median age (IQR) years** | 80 (68-87) |
| **OHCA Location (%)**  Home  Nurse facilities  Street  Public place  Workplace  Sport  Railway  Other | 1336 (84.5)  123 (7.8)  69 (4.4)  20 (1.3)  12 (0.8)  7 (0.4)  1 (0,1)  14 (0.9) |
| **Witnessed status (%)**  No  Yes bystander  Yes EMS  Unknown | 668 (42.3)  721 (45.7)  178 (11,3)  11 (0,7) |
| **Bystander CPR (%)**  No  Yes  Unknown | 1041 (66.1)  530 (33.7)  3 (0.2) |
| **Dispatch assisted CPR (%)**  No  Yes  Unknown | 1174 (75.6)  371 (23.9)  7 (0.5) |
| **Resuscitation attempted** | 1206 (76.2) |
| **Advanced resuscitation (%)^¶^** | 774 (64.2) |
| **ROSC^¶^** | 239 (19.8) |
| **Survived event (%) ^¶^** | 229 (19) |
| **Survival to hospital discharge (%) ^¶^** | 90 (7,4) |

IQR: interquartile range; OHCA: out-of-hospital cardiac arrest; CPR: cardiopulmonary resuscitation; ROSC: return of spontaneous circulation.

¶ includes only patients on whom resuscitation was attempted.
